# Low Threshold Voltage and Programmable Patterned Polymer-Dispersed Liquid Crystal Smart Windows

**DOI:** 10.3390/polym17182531

**Published:** 2025-09-19

**Authors:** Zhichao Ji, Zhenyuan Wang, Hongxu Jin, Xinying Cui, Meijun Liu, Tianzhen Chen, Lei Wang, Haibin Sun, Taoufik Soltani, Xinzheng Zhang

**Affiliations:** 1 College of Physics and Electronic Engineering, Xinyang Normal University, Xinyang 464000, China; wangzhenyuan1125@163.com (Z.W.); jinhongxu20@163.com (H.J.); cxying01282025@163.com (X.C.); liumeijun2025@163.com (M.L.); chentianzhen8259@163.com (T.C.); wdwanglei@xynu.edu.cn (L.W.); sunhaibin@xynu.edu.cn (H.S.); 2 The MOE Key Laboratory of Weak-Light Nonlinear Photonics and International Sino-Slovenian Join Research Center on Liquid Crystal Photonics, TEDA Institute of Applied Physics and School of Physics, Nankai University, Tianjin 300457, China; 3 LR99ES16 Laboratoire de Physique de la Matière Molle et de la Modélisation Electromagnétique, Faculté des Sciences de Tunis, Université de Tunis El Manar, Tunis 2092, Tunisia; tawfik_sol@yahoo.fr; 4 Collaborative Innovation Center of Extreme Optics, Shanxi University, Taiyuan 030006, China

**Keywords:** smart windows, PDLC, patterned PDLC, pre-orientation, low threshold voltage

## Abstract

Polymer-dispersed liquid crystal (PDLC) smart windows hold significant potential for energy-efficient buildings and vehicles, offering a promising pathway toward carbon neutrality. However, their widespread applications are hindered by critical limitations, including high driving voltages and the inability to achieve programmable patterning or multi-region addressable control. To address these challenges, we propose a pre-orientation strategy via low-voltage electric field (5 V, 1 kHz), which optimizes liquid crystal molecular alignment during the phase separation process. Vertically aligned liquid crystal molecules in the polymer network with enlarged pore structures reduce anchoring energy barriers for LC molecular reorientation, causing a 61.2% reduction in threshold voltage (*V*_th_) from 20.6 V to 8.0 V. Crucially, a programmable patterned PDLC film is successfully fabricated by utilizing cost-effective photomasks. Due to the different *V*_th_ of the corresponding regions, the patterned PDLC film exhibits stepwise control modes of light transmission: patterned scattering state, patterned transparent state and total transparent state, driven by incremental voltages. Our method can achieve not only energy-efficient tunable patterns for esthetic designs (e.g., logos or images) but also a scalable platform for multi-level optical modulation, which will advance PDLC technology toward low-voltage adaptive smart windows and open avenues for intelligent architectures and broadening their application scenarios.

## 1. Introduction

Smart windows based on polymer-dispersed liquid crystal (PDLC) technology have gained significant importance in building and automotive applications, aligning with global advancements in energy efficiency and carbon neutrality initiatives [1,2,3,4]. A typical PDLC film is composed of micro- or nano-sized liquid crystal (LC) droplets randomly dispersed in a continuous polymer matrix, formed via phase separation. By applying an external electric field, the reorientation of LC molecules allows dynamic modulation of optical transmittance, enabling dual functionality—privacy protection and radiative heat management [5,6,7]. Despite these advantages, conventional PDLC systems face practical challenges such as high driving voltages, limited contrast ratios (CR), and restricted optical switching modes. The high operating voltage mainly originates from the random orientation of LC droplets and strong anchoring effects at the polymer interface. In fact, the phase separation process is influenced by multiple factors including surface interactions, monomer viscosity, composition, curing temperature, and cell thickness [8,9]. Current optimization strategies focus on two main approaches: (1) incorporation of functional additives such as dichroic dyes [10,11], nanoparticles [12,13], or fluorinated compounds [14], and (2) modifying the polymer matrices through molecular engineering [9,15].

Beyond these methods, alignment control has emerged as a crucial factor affecting phase separation and final morphology of LC droplets [8,9]. For instance, surface alignment techniques (e.g., mechanical rubbing) can suppress LC droplet coalescence and promote uniformity, leading to improved response times and CR values. But this method exhibits a strong thickness dependence [16,17,18,19]. Alternatively, electric field-induced alignment offers a promising route to precisely control LC molecular orientation without requiring specialized alignment layers. This approach is particularly effective in systems containing polymerizable monomers, where in situ polymerization under an electric field permanently fixes the aligned structure, as demonstrated in several multicomponent systems [20,21,22,23,24,25]. Wang et al. reported that a high electric field pre-alignment during phase separation significantly enhanced electro-optical performance of the PDLC film by modifying LC–polymer interfacial interactions [26].

Conventional PDLC films are typically driven in a full-field mode, making it difficult to achieve patterned or stepwise transmittance control [27,28]. Recent efforts have focused not only on enhancing conventional PDLC performance but also on developing patterned PDLCs for emerging applications [29,30]. Notable methods include inkjet printing [31], photomask-assisted polymerization [32], and UV grayscale printing [33,34]. For example, Yan et al. demonstrated a triple-state patterned PDLC using grayscale photomasks, but OFF-state transmittance remained mask-dependent [33,34]. Wang et al. developed fluorescent dye/nanoparticle co-doped PDLC systems that simultaneously improved electro-optical performance and enabled patterned anti-counterfeiting applications [35]. Though these methods can realize the patterned PDLC, many critical limitations are still unsolved, including high driving voltages, complex material compositions, alignment layer dependencies, and extended UV exposure durations. Fundamentally, the inherent stochastic distribution of LC molecules in the droplets cannot be effectively regulated. Therefore, there is a pressing need for a simple, cost-effective strategy to fabricate step-driven patterned PDLCs with low driving voltage and high CR, alongside a deeper understanding of how pre-alignment governs electro-optical properties.

In this paper, we propose an electric field-assisted polymerization-induced phase separation (PIPS) strategy to engineer PDLC films with low *V*_th_ and programmable patterns. The effect of the field on the phase separation process is investigated systematically. Under a weak electric field, LC molecules align along the field direction, promoting the formation of an ordered polymer network. This synergistic alignment yields exceptional electro-optical performance, including an ultralow threshold voltage of 0.23 V/μm and a high contrast ratio exceeding 98:1, attributed to reduced interfacial anchoring. Combining with photomask-controlled multi-step polymerization, the ordered and unordered zones can be obtained, which permits spatial modulation of *V*_th_ and multilevel architectures (bi-/tri-state patterns) within a single film. The programmable pattern films enable step-driven transmittance control, including patterned scattering state, patterned transparent state and total transparent state. This field-guided alignment strategy not only surpasses conventional uniform PDLC films but also opens new avenues for energy-saving smart windows with customizable privacy zones.

## 2. Experimental Section

### 2.1. Materials

The desired polymer/LC composite materials were prepared by mixing a UV-curable optical adhesive (NOA65, Norland Products Inc, Cranbury, NJ, USA) and a positive nematic liquid crystal (E7, Nanjing Ningcui Optical Technology Co., Ltd., Nanjing, China). The mixtures, with E7:NOA65 weight ratios ranging from 7:3 to 5:5, were stirred at 700 rpm and heated at 70 °C on a hot plate for 3 h, respectively. LC cells without any alignment layer were fabricated using two cleaned indium tin oxide (ITO) glass substrates (P004, Zhuhai Kaivo Optoelectronic Technology Co., Ltd., Zhuhai, China) as upper and lower transparent electrodes. The cell gap was 30 μm. The heated mixture was injected into the LC cells at 70 °C. Subsequently, the samples were cooled naturally to room temperature. The refractive indices of E7 at room temperature are nₑ = 1.741 (extraordinary) and nₒ = 1.517 (ordinary) at 589 nm, and its clearing point is Tc = 59 °C. The refractive index of NOA65 (nₚ) is 1.524.

### 2.2. Methods

(1) Uniform PDLC films fabricated by two-step curing with/without electric field

**Figure 1:**
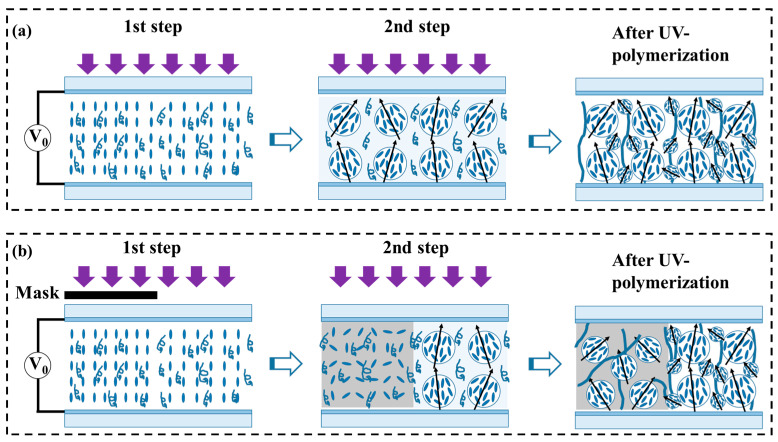
Schematic illustration of the two-step UV-polymerization procedures with/without AC applied voltage: (**a**) uniform PDLC film; (**b**) programmable patterned PDLC film.

As shown in Figure 1, a UV lamp (365 nm wavelength, Shenzhen Height-LED Opto-electronics Tech Co., Ltd., Shenzhen, China) operating at 26 mW/cm^2^ was employed to initiate the PIPS. To investigate the effects of the electric field on the phase separation process, a two-step polymerization method was employed. As shown in Figure 1a, the sample was first cured under a proper voltage (1 kHz, square wave) for 10 s to achieve partial polymerization of pre-oriented textures. And the LC molecular orientation could be precisely controlled through voltage adjustment. Secondly, continued curing without applied voltage for 6 min to enable complete phase separation. Due to the different polymerization conditions in each step, there are two distinct sizes of LC droplets within the resulting PDLC film. This sequential curing process ultimately produced uniform PDLC films exhibiting enhanced molecular ordering compared to conventional preparation methods.

(2) Programmable patterned PDLC film fabricated with a photomask and with/without electric field

To achieve the programmable patterned PDLC film, a custom-designed PET photomask was fabricated using a commercial label printer served as UV-absorbing templates, due to the carbon powder’s inherent optical attenuation properties. As illustrated in Figure 1b, in the first step, the photomask was placed on the sample surface. During UV irradiation, polymerization occurred selectively in the transparent areas under optimized electric field conditions (1 kHz square wave, 10 s duration), while the opaque areas remained free of polymerization. The duration time of the electric field applied during the first polymerization step was chosen based on the results shown in Figure S1. After removing the photomask and electric field, the sample was continually polymerized for 6 min to realize complete phase separation. Similarly to Figure 1a, in regions polymerized under an electric field, the liquid crystal droplets exhibit two distinct types of morphology: larger droplets with ordered alignment and smaller droplets with random orientation. In contrast, in areas polymerized without an applied electric field, the droplets are uniformly moderate in size and display entirely random orientation. Finally, the spatially patterned PDLC film with random and ordered alignment was obtained, indicating different optical modulation capabilities in different regions.

The composition and the curing conditions used for different samples were given in Table 1. Samples A1–A3 were cured under identical polymerization conditions without a pre-orientation electric field, while samples B1-B3 were cured under a 5 V, 1 kHz square wave.

### 2.3. Characterization

The morphology of the PDLC films was characterized using a scanning electron microscope (SEM, S-4800, Hitachi Limited, Tokyo, Japan). Before SEM observation, PDLC specimens underwent 48 h of acetone immersion for complete LC molecules extraction, then 10 h of ambient drying, and gold sputter coating to enhance surface conductivity. Electro-optic performance was evaluated using a homemade measurement setup consisting of a He-Ne laser (632.8 nm), a power sensor (OP-2 VIS, Coherent, Santa Clara, CA, USA), and an automated voltage control system (Agilent 33120A function generator coupled with a New Focus 3211 high-voltage amplifier). For measuring the response time, the power sensor was replaced by a high-speed photodetector (Det210, Thorlabs, Newton, NJ, USA), and the laser light intensity was converted into an electric signal and recorded by an oscilloscope (DPO 4054, Tektronix, Beaverton, OR, USA). To measure the transmission spectra, the samples were illuminated by using a deuterium–halogen white light source (DH-2000-BAL, Ocean Optics, Dunedin, FL, USA), and the transmission spectra were measured by coupling to a spectrometer (HR4000CG-UV-NIR, Ocean Optics, Dunedin, FL, USA).

The parameters for evaluating the electro-optical performance are defined below. Transmittance is the ratio of the transmitted light intensity from the sample to that from an empty LC cell. OFF-state transmittance (*T*_off_) denotes the transmittance of the PDLC without voltage applied. Maximum transmittance (*T*_on_) is the highest stable transmittance value achieved under increasing applied voltage. Both *T*_off_ and *T*_on_ are obtained directly from Voltage-Transmittance (V-T) curves. The CR defined as *T*_on_/*T*_off_, indicates the transmittance tunability. The threshold voltage (*V*_th_) and the saturation voltage (*V*_sat_) correspond to the voltages at 10% and 90% transmittance, respectively. Rise time is strongly dependent on the applied voltage and can be reduced by increasing the driving voltage. Fall time, often considered more critically than rise time, is typically the focus of study. OFF-state response time (*t*_off_) is defined as the time required for the transmittance to decrease from 90% to 10% without applied voltage. The parameters can be expressed by following equations [33,36]:

(1) $$V_{th}=\frac{d}{R}{\left[\frac{K\left(\omega^{2}-1\right)}{\omega_{0}∆\omega }\right]}^{1/2},$$

(2) $$V_{sat}=\frac{d}{R}{\left[\omega^{2}-1\right]}^{1/2}\frac{4\pi K}{∆\omega },$$

(3) $$t_{off}=\frac{\gamma R^{2}}{K(\omega^{2}-1)},$$

where *R*, *K*, *d*, *ω*, and *γ* represent the average LC domain size, elastic constant, film thickness, aspect ratio, and rotational viscosity of the LC, respectively.

## 3. Results and Discussion

### 3.1. Effect of the Electric Field During the Phase Separation

#### 3.1.1. Influence of Composite Ratio

**Figure 2:**
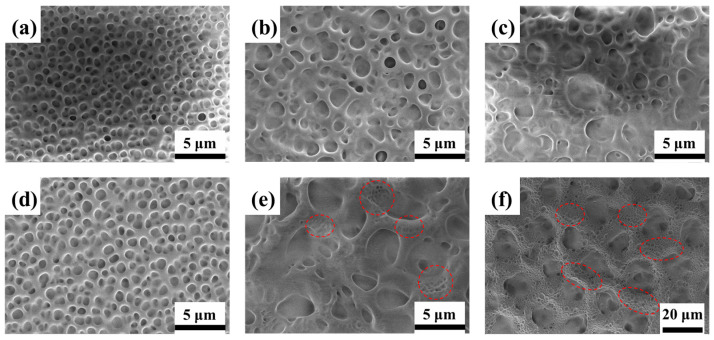
SEM photographs of uniform PDLC films with 0 V, 10 s for first step polymerization: (**a**) A1, (**b**) A2, (**c**) A3; uniform PDLC films with 5 V,10 s for first step polymerization: (**d**) B1, (**e**) B2, (**f**) B3.

The effect of the electric field-assisted PIPS depends on the component concentration of the pre-polymer/LC composite materials [37]. Figure 2 shows SEM micrographs of samples with different ratios and polymerization conditions, revealing their microstructure evolution mechanisms. The composite ratio significantly influences the pore size of the polymer network. As the LC ratio increases from 50% to 70%, the corresponding mesh size gradually increases. The decreased polymerizable monomer content leads to a sparser network during PIPS, resulting in larger LC droplets—a trend consistent with other reports [38]. Specifically, applying the pre-orientation voltage during the first step produced a larger polymer matrix pore size compared to samples with the same composite ratio cured without the field, particularly at high LC concentration. Furthermore, as the LC content increases, nanopores emerged within the polymer networks as indicated by the red dashed circles in Figure 2e,f. As can be seen in Figure S2, the average liquid crystal droplet size in sample A2 is 2.65 μm, while sample B2 exhibits a bimodal size distribution, with larger droplets measuring approximately 3 μm and smaller ones around 0.62 μm, respectively. During the first polymerization step, the electric field promotes phase separation of the LC under aligned conditions, while the short exposure time leads to incomplete phase separation and the formation of larger droplets. In the second step, further polymerization of the remaining monomers occurs in the absence of an electric field, inducing the formation of smaller, randomly oriented LC droplets. This change in matrix size demonstrates the pre-orientation voltage’s significant influence on the phase separation process.

**Figure 3:**
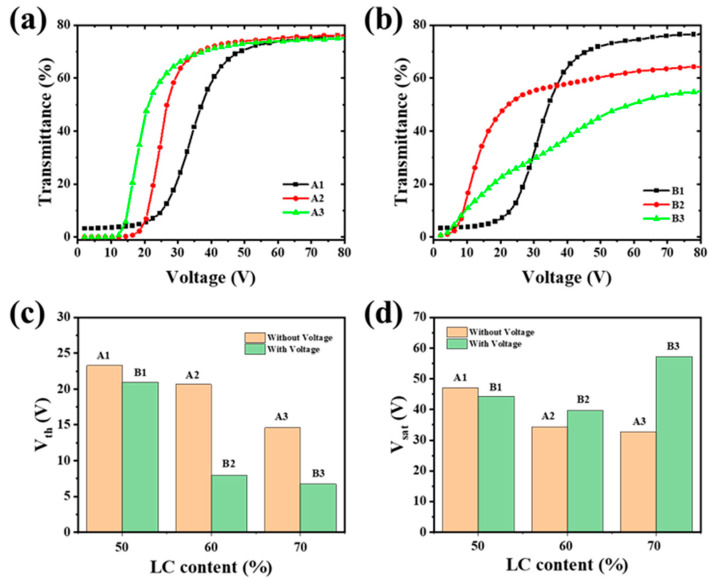
The V-T curve of samples polymerization (**a**) without, and (**b**) with pre-orientation electric field; (**c**) *V*_th_ and (**d**) *V*_sat_ of samples polymerization without/with the pre-orientation electric field.

The transmission changes in different samples are measured, Figure 3a shows the V-T curves for samples A1–A3. As the LC content increases from 50% to 70%, the *V*_th_ and *V*_sat_ gradually decreased. An increase in LC droplet size reduces the anchoring energy of the polymer matrix, which can be attributed to a weaker stabilization of LC molecules and lower confinement effects in large size droplets [36]. Figure 3b shows the V-T curves for samples B1–B3 polymerized with the applied electric field (5 V, 1 kHz). The curve of sample B1 keeps a similar contour to A1 due to the similar polymer network and LC droplet size (as shown in Figure 2a,d). But for B2 and B3, the curves are obviously different. Notably, compared to A3, the transmittance of sample B3 gradually increase with the applied voltage, lacking distinct threshold and saturation voltages. This behavior arises from the presence of dual-sized LC droplets within the polymer network, as evidenced in Figure 2f. According to Equations (1) and (2), the *V*_th_ and *V*_sat_ are inversely proportional to the LC droplet radius. Smaller droplets experience stronger surface anchoring forces, thereby requiring higher energy to reorient LC molecules from their initial alignment [28]. As the voltage increases, the LC droplets of different sizes progressively reorient sequentially, leading to the observed slow variation in transmittance. Despite variations in electro-optic response, all samples achieve reversible opaque-to-transparent switching upon voltage application.

The *V*_th_ and *V*_sat_ of samples A1–A3 and B1–B3 are presented in Figure 3c and 3d, respectively. As shown in Figure 3c, for the same LC content, PDLC films cured under the pre-orientation electric field exhibit reduced *V*_th_ values. Notably, when the weight ratio of E7 to NOA65 is 6:4, the voltage decreases by approximate 12.6 V. When the weight of E7 increase to 70%, the *V*_sat_ of sample B3 increases obviously, exhibiting a trend opposite to that of *V*_th_ as shown in Figure 3d. This may be caused by the two-step polymerization method. In the first step, the polymerization time was short, and the LC and polymer could not be totally separated. In the second step, residual polymer in composite materials continued to be crosslinked into polymer networks, and the LC droplet was smaller, causing the higher driving voltage. From the SEM graphs in Figure 2d–f, much smaller LC droplets appear in the polymer network. In the next experiment, we chose this ratio (LC:polymer = 6:4) as the appropriate concentration.

#### 3.1.2. Influence of Different Pre-Orientation Electric Fields with the Same Composite Ratio

**Figure 4:**
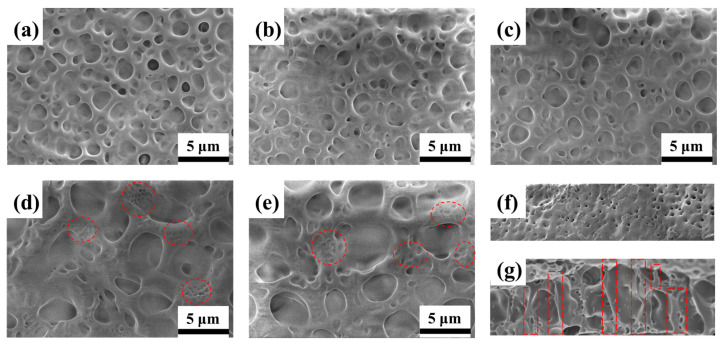
SEM photographs of uniform PDLC films with different pre-orientation electric fields during the first step polymerization: (**a**) A2, (**b**) C1, (**c**) C2, (**d**) B2, (**e**) C3; the cross-section of (**f**) A2, (**g**) B2.

To systematically investigate the impact of the pre-orientation electric field applied during phase separation on the electro-optical properties of PDLC films, uniform films were fabricated under different first-step curing voltages A2 (0 V), C1 (2 V), C2 (3 V), B2 (5 V), and C3 (10 V). For comparison, the samples cured at 0 V (A2) and 5 V (B2) were shown here again. Figure 4 shows SEM micrographs of samples A2, C1, C2, B2, and C3. Increasing the pre-orientation electric field during the first curing step results in a gradual enlargement of the polymer network mesh. Networks formed at lower voltages (≤3 V) exhibit random micron-sized pores embedded in a condensed polymer matrix. In contrast, sufficiently high pre-orientation fields produce networks characterized by sparse, large-sized pores surrounded by numerous nano-pores, as highlighted by the red circles. The structural evolution can be attributed to the dual role of the AC field during phase separation. First, field-induced perturbation promotes the coalescence of both LC and polymer domains, facilitating the formation of a sparser network with larger pores. Second, the electric field induces homeotropic alignment (perpendicular to the cell–substrate) of LC droplets, which persists after polymerization. As a result, the LC molecules adopt a more ordered arrangement in the OFF-state, thereby reducing the anchoring energy at the LC–polymer interface and consequently lowering the threshold voltage [26]. The enhanced structural order is clear when comparing the polymer networks in Figure 4g (B2, 5 V) and Figure 4f (A2, 0 V). Without an applied field, the porous structures are small and randomly distributed. In contrast, curing under an electric field promotes the formation of a polymer network with larger, more regular pores aligned along the homeotropic direction, as indicated by the red dashed boxes in Figure 4g. This electric-field-assisted phase separation mechanism differs fundamentally from rubbing alignment techniques, which typically produce smaller, more uniform LC droplets [17,18]. Finally, the microstructures observed in Figure 4d (B2, 5V) and Figure 4e (C3, 10V) exhibit similar characteristics.

**Figure 5:**
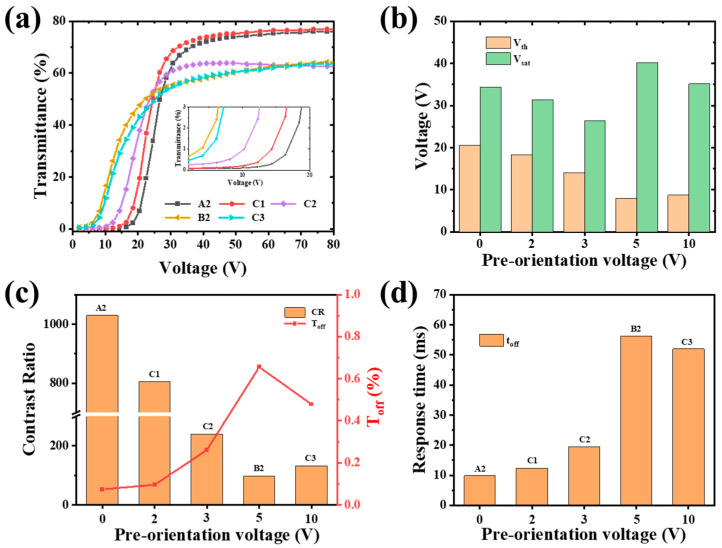
(**a**) Measured V-T curves; electro-optical properties (**b**) *V*_th_ and *V*_sat_, (**c**) CR and *T*_off_, (**d**) *t*_off_ with various pre-orientation voltages.

Figure 5 presents the electro-optical properties of samples prepared under different pre-orientation electric fields. The V-T curves for samples A2, C1, C2, B2, and C3 are shown in Figure 5a. As the pre-orientation voltage increased from 0 V to 5 V, the V-T curves shifted progressively toward lower driving voltages. Concurrently, the OFF-state transmittance exhibited a gradual increase, as clearly illustrated in the inset of Figure 5a. During the first polymerization step, the electric field promotes phase separation of the LC under aligned conditions, while the short exposure time leads to incomplete phase separation and the formation of larger droplets. Conversely, during the second polymerization step, further polymerization of the remaining monomers occurs in the absence of an electric field, inducing the formation of smaller, randomly oriented LC droplets, causing the ON-state transmittance exhibited a significant decrease. This indicates identifying the optimal pre-orientation voltage to balance the electro-optical performance of the device is important. Figure 5b demonstrates that the *V*_th_ decreased gradually from 20.6 V to 8.0 V. This reduction in *V*_th_ is attributed to the formation of larger and more orderly liquid crystal droplets within the PDLC films. However, at the higher pre-orientation voltage of 10 V (sample C3), *V*_th_ remained unchanged compared to that of sample B2 (prepared at 5 V). This stability is due to the similarity in the topography of the polymer network structure between samples C3 and B2, as confirmed in Figure 4d,e. Therefore, considering the significant *V*_th_ reduction achieved at 5 V and the lack of further improvement at 10 V, we selected 5 V as the optimal pre-orientation voltage. This demonstrates significant potential for energy-saving applications, and the improved electro-optical properties are superior to former results (shown in Supporting Information (Table S1) [26,28,31,32,35,39]. The variation trend of the *V*_sat_ is different from the *V*_th_. It decreases gradually as the pre-orientation voltages increase from 0 V to 3 V, caused by the larger LC droplet in PDLC films. When the voltage is 5 V, the *V*_sat_ becomes larger, resulting in the observation of smaller LC droplets in the polymer network. It is obvious that smaller LC droplets need higher driving voltage to achieve the reorientation of the LC molecules based on Equation (2).

**Figure 6:**
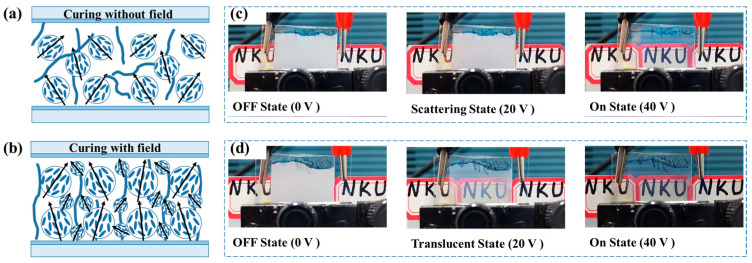
The diagram of the internal structure of PDLC film after curing without pre-orientation voltage (**a**), and with pre-orientation voltage (**b**), photographs of sample A2 (**c**) and sample B2 (**d**) in different states.

Figure 5c demonstrates that increasing the pre-orientation electric field during the first curing step leads to an increase in the *T*_off_ and a decrease in the CR of the PDLC films. This effect is likely attributed to the formation of larger and more ordered LC droplets, which enhance refractive index matching between the LC and polymer phases, thereby reducing the scattering of incident light at their interfaces [28]. Although the CR of sample B2 (cured at 5 V) decreases significantly to 98 compared to 1029 for sample A2 (cured at 0 V), it remains enough for effective privacy protection (as shown in Figure 6d). More importantly, this CR value is still higher than those achieved by other established methods, such as inkjet printing (4.4) [31], photomask-assisted polymerization (89.4) [32], and co-doping strategies (76.45) [35]. Therefore, B2, fabricated under an applied voltage of 5 V for 10 s, is identified as the optimal sample. Figure 5d indicates that *t*_off_ progressively increases with higher pre-orientation voltages. This delay is attributed to a weakened anchoring effect within the larger polymer network, which diminishes the restoring force. Consequently, upon removal of the external electric field, the LC molecules take longer time to return to their initial orientation state [40]. In contrast, films cured without a pre-orientation field exhibit a stronger anchoring effect, allowing the LC molecules to revert rapidly to their disordered state after field removal.

#### 3.1.3. Uniform PDLC Films Cured With/Without Electric Field

For the traditional UV-induced phase separation process, there was no alignment layer within the cell to orient the LC molecules. Consequently, the polymerized network was in a random state, and the resulting LC droplets were randomly distributed within the polymer network, as shown in Figure 6a. Under the same curing conditions, adjusting the LC/polymer weight ratio can modulate the LC droplet size.

When curing the polymer/LC composite materials under a pre-orientation electric field, LCs with positive dielectric anisotropy align along the field direction, and the molecular perturbations promote the separation between the LC and polymer. As shown in Figure 6b, the polymerized structure exhibits a more uniform distribution and larger LC droplets. Consequently, the interfacial anchoring force between LC molecules and the polymer network weakens, significantly reducing *V*_th_ as the pre-orientation voltage reaches 5 V. Furthermore, the ordered microstructure also reduces light scattering, yielding higher *T*_off_ and lower CR in the OFF-state. To enhance CR, a second curing step was implemented using prolonged UV irradiation without an electric field. Figure 6c shows sample A2 (cured without pre-orientation), achieving full transparency only at 40 V. In contrast, Figure 6d shows sample B2 (cured with 5 V, 10 s during time) achieves transparency at 20 V. While both samples maintain fundamental privacy protection in the OFF-state.

**Figure 7:**
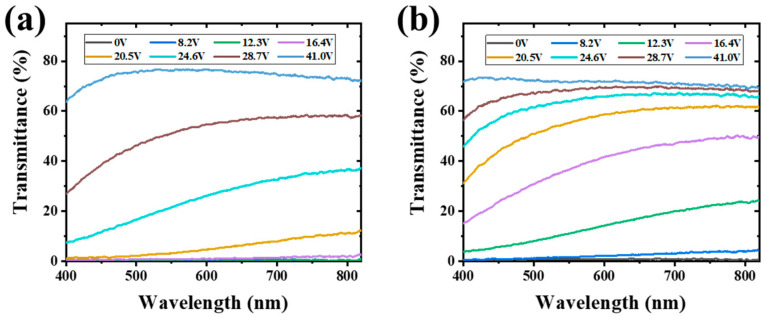
Transmittance curves of samples A2 (**a**) and B2 (**b**) at different voltages.

PDLC films enable dynamic privacy protection through voltage-driven reorientation of liquid crystal molecules, which modulates light transmittance. To evaluate their wavelength-dependent electro-optical behavior, we measured the transmittance spectra of samples A2 and B2 under progressively increasing drive voltages (Figure 7). Both samples showed strong OFF-state scattering, with transmittance below 1% across the entire visible spectrum. As the voltage increased to 8.2 V, sample B2 began to exhibit enhanced transmittance, whereas sample A2 remained largely opaque. Notably, the transmittance curves of A2 remained low and overlapping at 0 V, 8.2 V, and 12.3 V. Further voltage elevation led to a gradual increase in transmittance for both samples. Below 28.7 V, sample B2 consistently demonstrated higher transmittance than A2. These voltage-dependent transmittance differences highlight the capability for precise electro-optical control and suggest potential applications in step-driven patterned displays via spatial alignment modulation.

### 3.2. The Patterned PDLC Windows

#### 3.2.1. Patterned PDLC Windows Through Two-Step Polymerization with a Photomask

**Figure 8:**
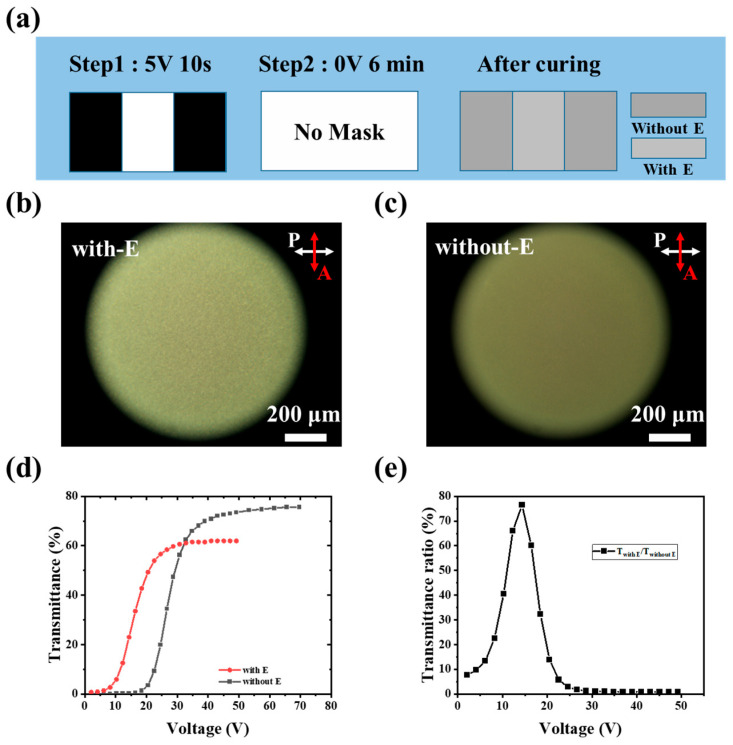
(**a**) The illustration of the photomask and polymerization process of the patterned PDLC film, the POM images of (**b**) Region with E and (**c**) Region without E in the OFF-state, (**d**) V-T curves of regions with E and without E, (**e**) the variation in the transmittance ratio of region with E and region without E under different voltages.

Based on the results discussed above, the *V*_th_ of the PDLC film can be tuned by the pre-orientation voltage. This finding indicates that differentiating individual regions within a single PDLC device is feasible, thus we proposed a two-step polymerization method utilizing a photomask (Figure 1b) to control the curing of different areas under distinct applied voltages. Figure 8a illustrates the photomask design and the polymerization conditions for each step. Specifically, in the first step, the sample was cured for 10 s under a pre-orientation voltage (5 V, 1 kHz, square wave) with the photomask. This resulted in the polymerization of the aligned LCs within the transparent areas of the mask (denoted as region with E). In the second step, the photomask and electric field were removed. Consequently, the LCs in the previously masked (opaque) areas reverted to a random orientation. The entire sample was then cured for 6 min to achieve complete polymerization. The region formed during this step, exhibiting random LC alignment, is designated as the region without E. This process yielded a single cell containing two distinct zones (with E and without E). As previously established, these zones exhibit different electro-optical properties due to distinct phase separation mechanisms, and the resulting PDLC film can be driven using fully covered electrodes. Figure 8b,c present polarized optical microscopy (POM) images of the region with E and region without E, respectively, in the OFF-state. The region without E appears darker than the region with E, attributable to its smaller LC droplets within the polymer network, which induce stronger scattering of incident light. Conversely, the brighter appearance of the region with E in Figure 8b signifies larger LC droplets within its polymer network.

**Figure 9:**
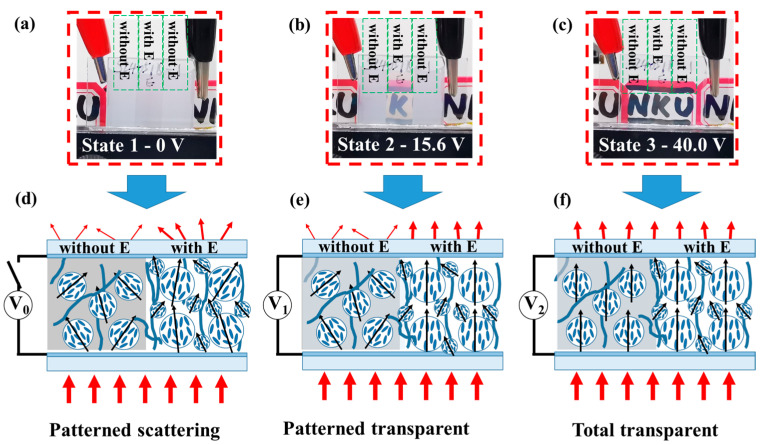
The photograph of patterned PDLC film under different applied voltages: (**a**) OFF-state (0 V), (**b**) patterned transparent state (15.6 V), (**c**) total transparent state (40.0 V); The illustration of LC molecules and the scattering effect of incident light under different states: (**d**) patterned scattering state, (**e**) patterned transparent state, and (**f**) total transparent state.

To investigate the electro-optical properties of these two distinct regions, their V-T curves were measured and presented in Figure 8d. The curve for the region with E (red line) was shifted leftward by approximately 10 V compared to a reference (likely the region without E), resulting in a lower threshold voltage (decreased from 21.85 V to 10.36 V) and a lower saturation voltage (decreased from 36.9 V to 24.02 V). Crucially, the presence of distinct electro-optical zones within the PDLC film demonstrates that its transmittance can be progressively controlled under a uniform applied electric field, enabling patterned display. Figure 8e illustrates the transmittance ratio between the two regions under the same driving voltage, highlighting the remarkable tunability of the patterned PDLC film. In the OFF-state, due to the different transmittance of two regions, the ratio is about 7.6, it can present a patterned display with privacy protection, this stands for the patterned scattering state as shown in Figure 9a, which is different from traditional uniform PDLC film. As the applied voltage increases, the transmittance ratio between the regions initially rises, reaches a maximum at approximate 15.6 V and then falls. At this voltage, the clearest patterned display is achieved: one zone becomes translucent while the other remains scattering. This state stands for a patterned transparent state as shown in Figure 9b. Upon further voltage increase (Figure 9c), both regions become highly transparent, causing the pattern to disappear, indicating the total transparent state.

#### 3.2.2. Experimental Mechanism Diagram

Figure 9 shows the photograph of patterned PDLC film at different drive voltages. Figure 9d–f shows the schematic diagram of LC molecules in the three states. The underlying mechanism is from different polymerization conditions. In region with E, due to the pre-orientation electric field, the LC droplets have a large and orderly distribution in the polymer network, causing the low *V*_th_. Conversely, in region without E, the LC droplets are small and random in the polymer network, causing the higher *V*_th_. When we increase the voltage to V_1_, the region with E allows the light passing through first, and the region without E still stop the light and presents the pattern. When the voltage continues to increase, the two regions both become transparent. So, using this method, the PDLC allows different regions to be driven step by step.

### 3.3. Programmable and Multilevel Patterned PDLC Films

#### 3.3.1. Programmable Logo

**Figure 10:**
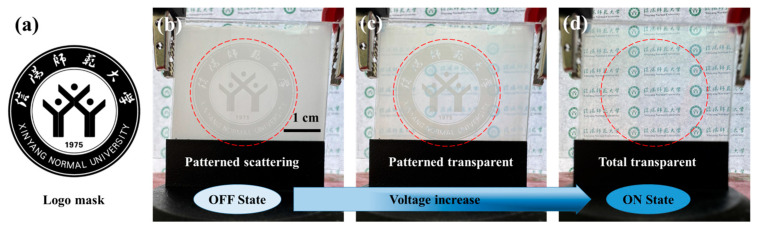
(**a**) The school emblem pattern of Xinyang Normal University; The photograph of different states of patterned PDLC film with the increase in the applied voltage, (**b**) patterned scattering state, (**c**) patterned transparent state, (**d**) total transparent state.

To further demonstrate the feasibility of our method, we designed a school emblem pattern of Xinyang Normal University (Figure 10a). In this experiment, a transparent region was first exposed for 10 s under a 5 V electric field. Subsequently, both the electric field and photomask were removed, and UV exposure continued for 6 min. This process yielded a pattern consistent with the photomask. In the OFF-state (no applied field), the device exhibits the patterned scattering state with privacy protection, owing to the different *T*_off_. When an increasing electric field is applied, the region pre-cured under the orienting field turns to a translucent state. The region cured without the electric field remains scattered (Figure 10c). As the field strength further increases, the latter region also changes to a translucent state. In the fully ON-state, both regions become transparent, causing the pattern to disappear. Moreover, upon decreasing the electric field, the device reverts to its initial scattering state due to the anchoring effect of the polymer network. A movie demonstrating this behavior is provided in Supplementary Materials (Video S1).

#### 3.3.2. Multilevel Pattern

**Figure 11:**
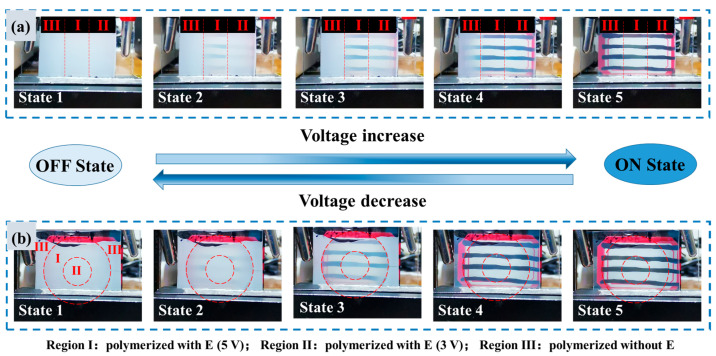
The photograph of different states of patterned PDLC film with applied voltage increase/decrease: (**a**) three-grid pattern, (**b**) concentric ring pattern.

Figure 11 displays multilevel patterned PDLC films comprising three distinct regions: I, II, and III. The preparation procedure was as follows. Step 1: Region I was polymerized under a 5 V applied voltage for 10 s, while regions II and III were shielded by the photomask. Step 2: Region I and II were polymerized under a 3 V applied voltage for 10 s, with region III remaining shielded by the photomask. Final Step: The voltage and photomask were removed, and the entire cell underwent continuous polymerization for 6 min to ensure enough phase separation. Three-grid and concentric ring structures were fabricated separately. Based on the V-T curves in Figure 5a, the three regions possess distinct *V*_th_, enabling stepwise control of their optical states. As the applied voltage increases, the regions sequentially transition from a scattering state, through a translucent state, to a transparent state, resulting in a dynamic patterned display. When decreasing the voltage, the device reverts to its initial scattering state. Detailed demonstrations of these tunable behaviors are provided in Supplementary Materials (Videos S2 and S3).

## 4. Conclusions

In summary, we propose an electric field-assisted phase separation method that is both energy-efficient and effective. Utilizing this approach, the electro-optical performance of PDLC films is enhanced, achieving a low threshold voltage of 0.23 V/μm and a contrast ratio of 98 under a pre-orientation voltage of 5 V. Specifically, by adjusting the pre-orientation electric field, the electro-optical performance can be tuned rather than by altering the chemical composition. By combining a photomask with the multi-step polymerization method, the programmable patterned PDLC films can be fabricated. These films support at least three voltage-controlled states: the patterned scattering state, patterned transparent state, and total transparent state. Furthermore, we systematically investigated the role of the pre-orientation electric field during phase separation and the mechanism of step-driven patterned display. The electric field pre-orients LC molecules and modulates LC droplet size, thereby determining the electro-optical properties. Based on this mechanism, visually complex designs, logos, or images with multiple grayscale levels can be achieved, significantly broadening potential applications in energy-efficient buildings and vehicles.

## Figures and Tables

**Table 1:** The component ratios and the curing conditions of PDLC film samples.

Sample	E7 (wt. %)	NOA65 (wt. %)	1st Step	2nd Step
A1	50	50	0 V, 10 s	0 V, 6 min
A2	60	40	0 V, 10 s	0 V, 6 min
A3	70	30	0 V, 10 s	0 V, 6 min
B1	50	50	5 V, 10 s	0 V, 6 min
B2	60	40	5 V, 10 s	0 V, 6 min
B3	70	30	5 V, 10 s	0 V, 6 min
C1	60	40	2 V, 10 s	0 V, 6 min
C2	60	40	3 V, 10 s	0 V, 6 min
C3	60	40	10 V, 10 s	0 V, 6 min

## Data Availability

The data that support the findings of this study are available from the corresponding author upon reasonable request.

## Supplementary Materials

The following supporting information can be downloaded at: https://www.mdpi.com/article/10.3390/polym17182531/s1, Figure S1: the school emblem patterned PDLCs fabricated with different electric field durations during the first polymerization step; Figure S2: Percentage distribution of droplet sizes for samples A2 and B2; Table S1: Comparison of electro-optical properties of PDLC devices fabricated with different techniques; Video S1: the different states of patterned PDLC film with the increase or decrease in the applied voltage; Video S2: the different states of three-grid pattern PDLC with applied voltage increase/decrease; Video S3: the different states of concentric ring pattern PDLC with applied voltage increase/decrease.
