# Supplementary material for: Low Threshold Voltage and Programmable Patterned Polymer-Dispersed Liquid Crystal Smart Windows
Source: Polymers (Basel). 2025 Sep 19;17(18):2531. doi: 10.3390/polym17182531 (PMC12473949; doi:10.3390/polym17182531)
Supplement: Supplementary file 1 [file polymers-17-02531-s001.zip › polymers-3836243-Supporting information-done.pdf]

polymers

MDPI

Supporting Information

Low Threshold Voltage and Programmable Patterned Polymer-Dispersed Liquid Crystal Smart Windows

Zhichao Ji [1,2,*], Zhenyuan Wang [1], Hongxu Jin [1], Xinying Cui [1], Meijun Liu [1], Tianzhen Chen [1], Lei Wang [1], Haibin Sun [1], Taoufik Soltani [3] and Xinzheng Zhang [2,4,*]

1 College of Physics and Electronic Engineering, Xinyang Normal University, Xinyang 464000, China; wangzhenyuan1125@163.com (Z.W.); jinhongxu20@163.com (H.J.); cxying01282025@163.com (X.C.); liumeijun2025@163.com (M.L.); chentianzhen8259@163.com (T.C.); wdwanglei@xynu.edu.cn (L.W.); sunhaibin@xynu.edu.cn (H.S.)

2 The MOE Key Laboratory of Weak-Light Nonlinear Photonics and International Sino-Slovenian Join Research Center on Liquid Crystal Photonics, TEDA Institute of Applied Physics and School of Physics, Nankai University, Tianjin 300457, China

3 LR99ES16 Laboratoire de Physique de la Matière Molle et de la Modélisation Electromagnétique, Faculté des Sciences de Tunis, Université de Tunis El Manar, Tunis 2092, Tunisie; tawfik_sol@yahoo.fr (T.S.)

4 Collaborative Innovation Center of Extreme Optics, Shanxi University, Taiyuan 030006, China

* Correspondence: jizc@xynu.edu.cn (Z.J.); zxz@nankai.edu.cn (X.Z.)

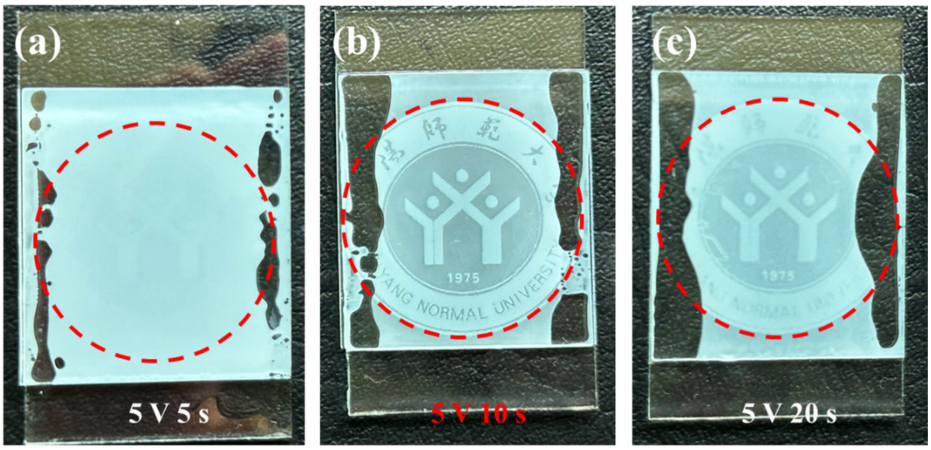

Figure S1. the school emblem patterned PDLCs fabricated with different electric field durations during the first polymerization step.

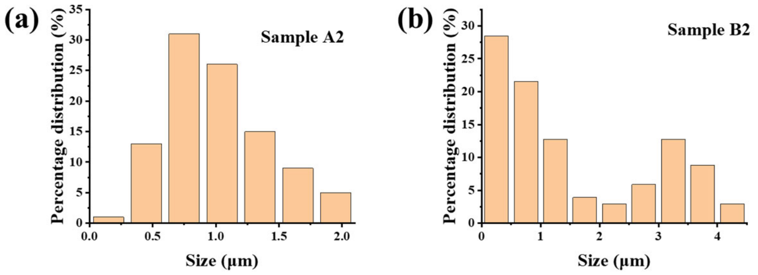

Figure S2. Percentage distribution of droplet sizes for samples A2 and B2.

Table S1. Comparison of Electro-Optical Properties of PDLC Devices Fabricated with Different Techniques.

Different PDLC technology	V_{th}	CR	OFF-state response time	Year [Ref. No.]
Inkjet printing	0.7 V/μm	4.4	77 ms	2022 [1]

 https://doi.org/10.3390/xxxxx

Photo-mask-assisted polymerization	0.9 V/μm	89.4	173.95 ms	2023 [2]
UV grayscale printing	0.8 V/μm	----	15.62 ms	2022 [3]
Co-doping strategy	0.77 V/μm	76.45	58.90 ms	2024 [4]
Electric field-assisted PIPS	0.23 V/μm	98	56 ms	this work

[1] Kamal, W.; Li, M. M.; Lin, J. D.; Parry, E.; Jin, Y. H.; Elston, S. J. A. A. Castrejón-Pita, S. M. Morris, Spatially Patterned Polymer Dispersed Liquid Crystals for Image-Integrated Smart Windows. *Adv. Opt. Mater.* **2022**, *10*, 2101748.

[2] Chen, G.; Zhang, J.; Ren, Y.; Sun, C.; Hu, W.; Yang, H.; Yang, D. K. Programmable Patterned Dynamic Scattering of Liquid Crystals/Epoxy-based Polymer Composite Film Sandwiched between Two Fully Covered Electrodes. *Adv. Opt. Mater.* **2023**, *11*, 2300537.

[3] Yan, J.; Fan, X. W.; Liu, Y. F.; Yu, Y.; Fang, Y. M.; Li, R. Z. Passive patterned polymer dispersed liquid crystal transparent display. *Chin. Opt. Lett.* **2022**, *20*, 013301.

[4] Lu, Y. F.; Yang, D. L.; Gao, H.; Du, X.; Zhao, Y. Z.; Wang, D.; He, Z. M.; Miao, Z. C.; Cao, H.; Yang, Z.; He, W. L.; Li, Y. Z. Enhanced electro-optical properties of polymer-dispersed liquid crystals co-doped with fluorescent molecules and nanoparticles for multifunctional applications. *Chem. Eng. J.* **2024**, *485*, 149654.
